# Structural, Functional, and Bioactive Properties of Sulfated Polysaccharides from Skipjack Tuna Skin as a Function of Drying Techniques

**DOI:** 10.1002/gch2.202400083

**Published:** 2024-08-03

**Authors:** Shahab Naghdi, Masoud Rezaei, Mehdi Alboofetileh, Mehdi Tabarsa, Mehdi Abdollahi, Jamshid Amiri Moghaddam

**Affiliations:** ^1^ Seafood Processing Department Marine Sciences Faculty Tarbiat Modares University Noor 46414‐356 Iran; ^2^ Fish Processing Technology Research Center Iranian Fisheries Sciences Institute Agricultural Research Education and Extension Organization (AREEO) Bandar Anzali FF7C+9X9 Iran; ^3^ Department of Life Sciences–Food and Nutrition Science Chalmers University of Technology Gothenburg SE 412 96 Sweden; ^4^ Chemical Biology Leibniz Institute for Natural Product Research and Infection Biology e.V. Hans‐Knöll‐Institute Beutenbergstraße 11a 07745 Jena Germany

**Keywords:** antibacterial properties, antioxidant activities, drying methods, *Katsuwonus pelamis*, sulfated polysaccharides

## Abstract

The study aims to investigate the impact of various drying techniques on the quality of sulfated polysaccharides (SP) extracted from Skipjack tuna (*Katsuwonus pelamis*) skin. Three drying methods, namely microwave drying (M‐KPP), freeze‐drying (F‐KPP), and hot air drying (HA‐KPP), are examined. The chemical and monosaccharide compositions of SP are significantly affected by the drying methods. The extraction yields for M‐KPP, F‐KPP, and HA‐KPP are 3.30%, 3.11%, and 2.50%, respectively (P < 0.05). Additionally, HA‐KPP, with 10.67% moisture content, exhibits the lowest moisture level among the dried samples (P < 0.05). Furthermore, the structural properties of SP remain consistent across different drying methods, as indicated by FTIR, XRD, and DSC analyses. F‐KPP demonstrates the highest antioxidant properties. The functional and antimicrobial activities of SP are significantly influenced by the drying technique, with hot air drying resulting in increased foaming capacity and microwave drying showing enhanced antimicrobial activity. In conclusion, the findings demonstrate that the functionality and bioactivity of SP from tuna skin are greatly influenced by the drying technique employed, suggesting that the selection of the optimal method should be tailored to the desired properties of the SPs and given careful consideration.

## Graphical abstract

The paper reports the impact of various drying techniques on the quality of sulfated polysaccharides (SP) extracted from Skipjack tuna skin. Functional and antimicrobial activities of SP are significantly influenced by the drying technique, with hot air drying resulting in increased foaming capacity and microwave drying showing enhanced antimicrobial activity.

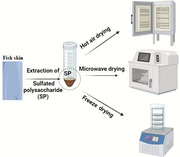

## 1 Introduction

Seafood products and aquaculture are crucial in meeting the global demand for protein‐rich food.^[^ ^1^ ^]^ In 2020, 178 million tons of aquatic animals were produced in the world.^[^ ^2^ ^]^ It is important to highlight that fish by‐products, including viscera, skin, head, and bones, typically make up ≈30 to 60% of the initial weight of the raw material, which has raised environmental and economic concerns globally.^[^ ^3^ ^]^ Therefore, utilizing these resources to extract bioactive compounds, such as sulfated polysaccharides (SPs), can be seen as an efficient and promising solution.

SPs are heterogeneous groups of polymeric carbohydrate molecules with a sulfate group attached to their hydroxyl group, which possess a wide range of biological activities such as antibacterial,^[^ ^4^ ^]^ antivirus,^[^ ^5^ ^]^ immune enhancing,^[^ ^6^ ^]^ antitumor,^[^ ^7^ ^]^ and antioxidant activities.^[^ ^7^ ^]^ The extraction of these compounds from marine animals involves enzymatic hydrolysis of the sample, followed by precipitating the obtained extract from the hydrolysis step using ethanol or CPC to isolate the polysaccharides.^[^ ^3^, ^4^ ^]^ Then, the isolated polysaccharides are typically dried using a freeze dryer and then stored.^[^ ^3^, ^4^ ^]^ Today, despite the significant impact of the drying method on the final samples, this aspect has not received sufficient attention. In addition, the majority of research conducted in this field far has primarily focused on examining the impact of various drying methods on initial samples or derived polysaccharides from plants and marine mollusks.^[^ ^8^ ^]^

Based on the information provided, there is a lack of knowledge regarding the impact of alternative drying methods on the structure and quality of sulfated polysaccharides derived from fish and seafood by‐products. Therefore, this study was aimed to investigate the effects of hot air drying, freeze‐drying, and microwave drying techniques on the structural, functional (emulsification and foaming activity), antioxidant, and antimicrobial properties of SPs isolated from Skipjack tuna skins.

## 2 Results and Discussion

### 2.1 Chemical and Monosaccharide Compositions of SPs

The proximate compositions of the used skin sample showed that its moisture, fat, protein and ash content were 62.06, 6.98, 27.62, and 2.56%, respectively. The results of the chemical composition, moisture content and extraction yield of SPs dried by different methods are presented in **Table** **1**. The samples displayed varying levels of moisture content, with HA‐KPP containing the least amount at 10.67% and M‐KPP containing the highest at 14.00%. The lipid content of F‐KPP was lower than others with significant differences (P < 0.05). The yield of M‐KPP (3.30%) was significantly higher than those of F‐KPP (3.11%) and HA‐KPP (2.50%) (P < 0.05), indicating that microwave drying serves as a better method for the drying of sulfated polysaccharide from skipjack tuna skins. This could be because the strong heat produced by the microwave leads to a significant increase in vapor pressure and internal temperature within the plant tissue, causing the breakdown of plant cell wall polymers.^[^ ^9^, ^10^ ^]^ Similar results have also been obtained in previous studies.^[^ ^9^, ^10^ ^]^ However, the different results were reported by Shang et al.^[^ ^11^ ^]^ in which the freeze‐drying technique showed the best results in drying polysaccharides from *Silphium perfoliatum* L. The lowest carbohydrate content (45.44 ± 1.91%) was in M‐KPP, while the highest was observed in the sample dried with HA‐KPP (51.56 ± 1.31%), and F‐KPP had 50.17 ± 0.95% of carbohydrate. The protein content in different samples showed a significant difference, and in this regard, the highest protein content of 16.80% was recorded in F‐KPP (p < 0.05). Interestingly, there was no significant difference found in the sulfate content of the samples, which ranged from 8.05% to 9.06%. However, F‐KPP had the highest sulfate content. The highest uronic acid content (4.26 ± 0.19) was found in F‐KPP and did not show any significant difference compared to the others. The findings of Shang et al.^[^ ^12^ ^]^ indicate that polysaccharides dried by the freeze‐drying method were higher in uronic acid and sulfate than others, which is consistent with the present research. Because of this phenomenon, freeze‐drying may be the most effective method for removing moisture and obtaining the highest amounts of polysaccharides. Further, Ma et al.^[^ ^13^ ^]^ demonstrated that freeze‐drying polysaccharides resulted in the highest protein and uronic acid content. These variations can be attributed to various environmental factors, such as the oxygen level and temperature employed during the drying process.^[^ ^14^, ^15^ ^]^ It has been well‐documented that the drying methods used for polysaccharides can significantly impact their chemical composition, leading to alterations in their bioactivity and functional properties.^[^ ^16^ ^]^ Additionally, it is possible that the presence of vacuum and oxygen during the drying process could lead to the degradation or destruction of the polysaccharide constituents.^[^ ^17^ ^]^ The research conducted by Shang et al.^[^ ^16^ ^]^ revealed that polysaccharides dried through freeze‐drying exhibited higher levels of uronic acid and sulfate compared to other methods, which aligns with the present study. Consequently, freeze‐drying may be considered the most effective technique for eliminating moisture and obtaining the highest quantities of polysaccharides. Additionally, Ma et al.^[^ ^13^ ^]^ demonstrated that freeze‐drying polysaccharides yielded the highest protein and uronic acid content.

**Table 1:** Chemical and monosaccharide compositions of dried sulfated polysaccharides from Skipjack tuna skin using different drying methods.

	M‐KPP	F‐KPP	HA‐KPP	Raw material
Chemical composition				
Yields (%)	3.30 ± 0.09^a^	3.11 ± 0.18^a^	2.50 ± 0.04^b^	–
Total sugars (%)	45.44 ± 1.91^b^	50.17 ± 0.95^a^	51.56 ± 1.3^a^	–
Total proteins (%)	13.13 ± 0.64^c^	16.80 ± 0.32^a^	15.60 ± 0.35^b^	27.62 ± 0.60
Uronic acid (%)	4.17 ± 0.15^a^	4.26 ± 0.19^a^	3.98 ± 0.11^a^	–
Sulfate (%)	8.05 ± 0.64^a^	9.06 ± 0.23^a^	8.26 ± 0.30^a^	–
Moisture Content (%)	14.00 ± 0.82^a^	12.33 ± 0.94^b^	10.67 ± 0.47^c^	62.06 ± 0.06
Lipid (%)	1.14 ± 0.14^a^	0.79 ± 0.07^b^	0.93 ± 0.03^b^	6.98 ± 0.24
Ash (%)	1.74 ± 0.03^a^	1.32 ± 0.07^a^	1.86 ± 0.07^a^	2.56 ± 0.36
Monosaccharide composition				
Rhamnose (%)	16.2	16.3	15.9	
Xylose (%)	15.6	16.2	15.8	
Mannose (%)	17.4	17.5	16.4	
GlcA (%)	24.1	24.3	25.8	
GalA (%)	25.7	26.7	26.1	

GlcA (glucuronic acid) and GalA (galacturonic acid). Data are calculated based on wet weights. Different letters in the same raw indicate significant differences (p < 0.05). ⁎ % of dry weight.

Table 1 displays the monosaccharide composition of the dried sulfated polysaccharides (SPs). The result shows that all samples have a similar monosaccharide profile, including Rhamnose, Xylose, Mannose, GlcA (glucuronic acid), and GalA (galacturonic acid). Although there may be slight variations in the content of these monosaccharides among the different polysaccharides, they share a common profile. However, it is worth noting that the freeze‐drying treatment appears to increase the xylose content in the dried SPs when compared to M‐KPP and HA‐KPP samples. This may be due to the potential oxidation of hydroxyl groups and the disruption of intermolecular hydrogen bonds that can occur when polysaccharides are dried in an oxygen‐rich environment or at high temperatures. These processes can affect the monosaccharide composition and lead to changes such as an increase in xylose content.^[^ ^14^ ^]^ Also, the high content of GalA observed in F‐KPP is consistent with the higher content of uronic acids in this polysaccharide.^[^ ^15^ ^]^ However, it can be deduced that the drying methods used had minimal impact on the constituent monosaccharides of the dried SPs. This finding is consistent with the findings of Fu et al.,^[^ ^18^ ^]^ who conducted a study comparing the structural characteristics and bioactivity of polysaccharides derived from loquat leaves using different drying methods. They observed that the monosaccharide composition remained unchanged regardless of the drying techniques employed. Similarly, Shang et al.^[^ ^16^ ^]^ investigated the impact of drying methods on the physicochemical properties and antioxidant activities of polysaccharides from *Astragalus* and discovered that all three polysaccharides exhibited consistent monosaccharide compositions. Liu et al.^[^ ^19^ ^]^ and Zou et al.^[^ ^20^ ^]^ also suggested that different drying methods did not lead to variations in the types of monosaccharides present in dried polysaccharides, but rather influenced the molar ratios of these monosaccharides.

### 2.2 Molecular Weight

**Figure 1:**
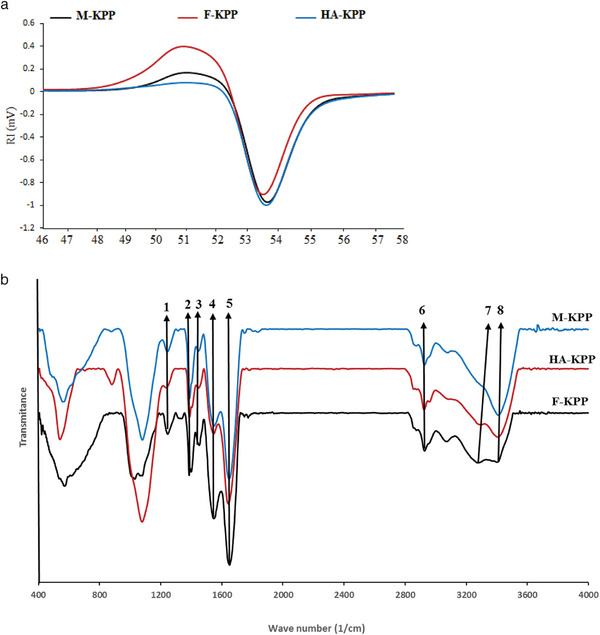
RI chromatograms a) and FT‐IR Spectra b) of dried sulfated polysaccharides from Skipjack tuna skin using different drying methods.

**Figure** **1**a depicts the RI chromatograms for dried SPs. As shown, all dried SPs exhibited a single peak at an elution time of 50 min, indicating that F‐KPP, HA‐KPP, and M‐KPP had molecular weights of ≈18.9, 28.3, and 19.95 kDa, respectively. These results indicate that the dried SPs had a consistent weight distribution. However, these findings were lower than those reported by Jridi et al.,^[^ ^4^ ^]^ who extracted SPs from Bullet tuna (*Auxis Rochei*) by‐products using an enzymatic method. They reported multiple peaks in the molecular weight distribution diagram of the extracted SPs. It has been noted that the molecular weight of polysaccharides can vary depending on the extraction process, purification techniques, and deproteinization treatment.^[^ ^21^, ^22^ ^]^ By the way, Liu et al.^[^ ^19^ ^]^ reported that different drying methods significantly influenced the molecular weight distributions of polyphenolic‐protein‐polysaccharide conjugates from *Hovenia dulcis*. A similar result was also observed for polysaccharides isolated from loquat leaves, suggesting that the polysaccharides rapidly aggregated during the drying process at relatively high temperatures.^[^ ^18^ ^]^ The findings of our study revealed that the molecular weight of freeze‐dried polysaccharides was higher compared to other samples. This could be attributed to the fact that an increase in temperature during the drying process can lead to a decrease in the molecular weight of the samples. In support of our results, Li et al.^[^ ^15^ ^]^ conducted a study where they observed similar outcomes. They found that samples dried using the freeze‐drying method had a higher molecular weight compared to other drying methods such as vacuum drying, microwave drying, hot air drying, and radio frequency drying. However, contrasting results have been observed with regards to the aforementioned materials.^[^ ^14^ ^]^ These results suggest that polysaccharide molecules have a greater tendency to aggregate at relatively high temperatures. This aggregative effect may be attributed to removing some of the hydration layer during the drying process, which compromises the structural integrity of the polysaccharides and promotes aggregation.^[^ ^16^ ^]^ Furthermore, it is also reported that the combination of high temperatures and shear forces in the spray‐drying technique can potentially disrupt the polysaccharide structure and lead to re‐aggregation.^[^ ^23^ ^]^

### 2.3 Structural Characterization of SPs

#### 2.3.1 FT‐IR Spectra

FT‐IR spectroscopy was performed in the 400–4000 cm^−1^ range to analyze the functional groups present in the dried‐SPs, and the results are displayed in Figure 1b. Furthermore, **Table** **2** indicates the peak number assigned to the functional group of each sample. As shown in Figure 1b and Table 2, there were some variations in the spectra of the dried‐SPs; however, the main bands were observed consistently across all SPs. The bands at ≈3250, 2850, 1620, and 1520 cm^−1^ correspond to amide A, amide B, amide‐I, and amide‐II, respectively. Similar spectra have been reported by Jridi et al.^[^ ^4^ ^]^ and Abdelhedi et al.^[^ ^24^ ^]^ for polysaccharides extracted from *Auxis Rochei* by‐products (skin, bone, head) and *Mustelus mustelus* viscera, respectively. The amide A and B regions exhibited typical bonds for alkyl and hydroxyl functional groups. Two absorbance peaks at 1383 and 1457 cm^−1^ were associated with uronic acids (O═C─O bending), which aligns with the findings of Souissi et al.^[^ ^22^ ^]^ The C═O band of uronic acids, which is a characteristic of the primary constituent of glycosaminoglycans, was observed at the amide I band (1650 cm^−1^), displaying similar intensities across all dried‐SPs.^[^ ^4^ ^]^ Additionally, the presence of ester sulfate groups (S─O) was indicated by a vibration band ≈1250 cm^−1^, as reported by Jridi et al.^[^ ^4^ ^]^ and Yang et al.^[^ ^25^ ^]^ Although this band appeared at the same wavelength for all dried‐SPs, F‐KPP exhibited the highest peak intensity, confirming its high sulfated group content.^[^ ^4^ ^]^ Previous studies investigating the effects of various drying methods on the properties of plant polysaccharides have demonstrated that the drying process, particularly the temperature, reduces the water content, leading to a decrease in the intensity of the hydroxyl peak.^[^ ^26^, ^27^ ^]^ However, in our study, there was no important change in the intensity of the O─H band among the samples. These findings are consistent with the results reported by Hu et al.,^[^ ^8^ ^]^ who examined the effects of different drying methods (freeze‐drying, spray‐drying, and rotary evaporation‐drying) on the physicochemical properties and antioxidant activities of polysaccharides from *Crassostrea gigas*. Similarly, Fu et al.^[^ ^29^ ^]^ reported alike findings, where dried polysaccharides extracted from loquat leaves using different drying methods such as freeze drying, hot‐air drying, vacuum drying, and microwave drying did not exhibit any significant impact on the FTIR spectra of the dried samples.

**Table 2:** Assignments of main peaks in the FTIR spectra of dried samples (Wavenumber in 1 cm^−1^).

Peak number	Peak assignment	M‐KPP	H‐KPP	F‐KPP
1	S─O	1245.85	1240.07	1245.85
2	O─C═C	1386.64	1386.64	1386.64
3	─CO	1429.06	1452.21	1450.28
4	Amide‐II	1550.56	1544.78	1550.56
5	Amide‐I	1650.85	1645.06	1637.35
6	Amide‐B	2927.56	2927.56	2927.56
7	Amide‐A	3299.77	3286.27	3280.48
8	OH	3398.13	3408.06	3409.53

#### 2.3.2 Differential Scanning Calorimetry (DSC)

**Figure 2:**
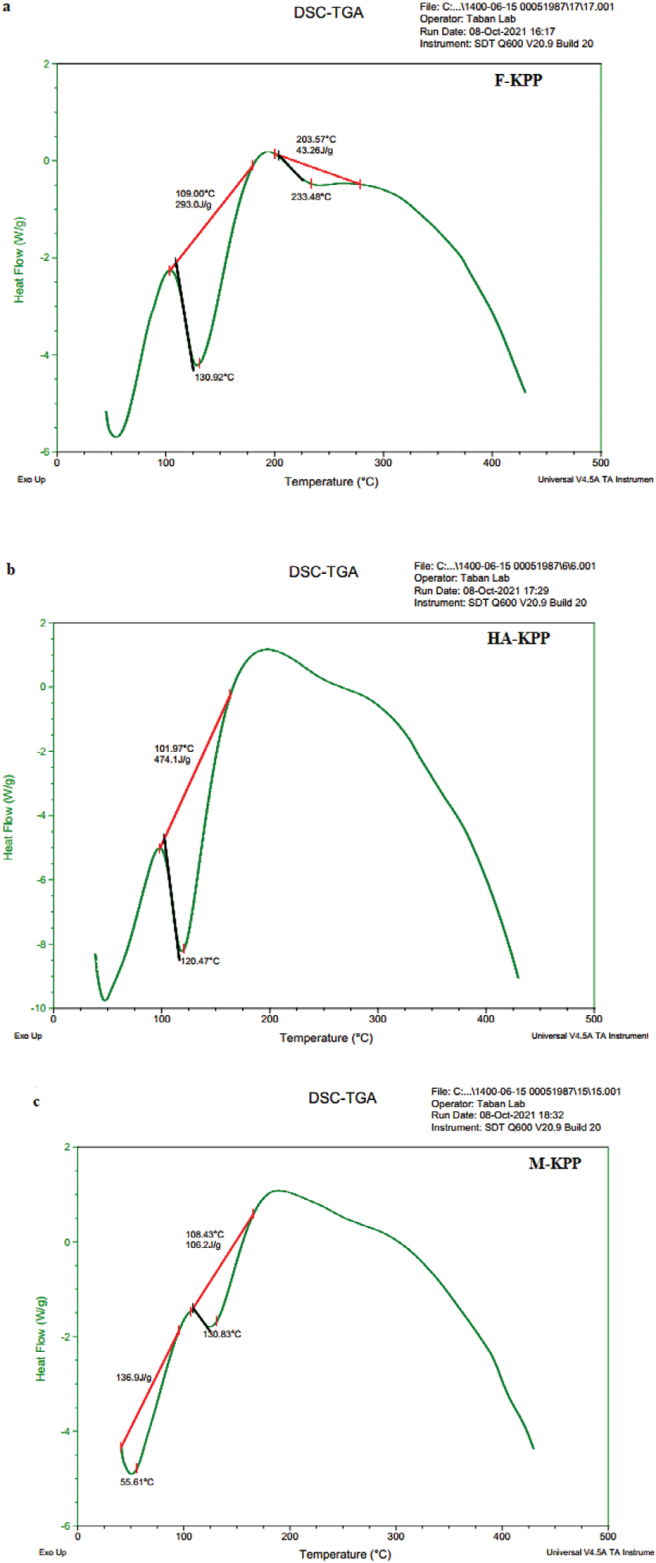
DSC thermographs of dried sulfated polysaccharides from Skipjack tuna skin using different drying methods including freezing dried‐SP (a), hot air dried‐SP (b), microwave dried‐SP (c).

DSC analysis is a valuable tool for studying the thermal behavior of polysaccharides at different temperatures Liu et al.^[^ ^30^ ^]^ The results of the DSC analysis are presented in **Figure** **2**, showing that the dried samples exhibited similar behavior. In all dried SPs, the first peak in the DSC graph appeared ≈100 °C, indicating the loss of adsorbed and structural water in the biopolymers.^[^ ^8^, ^28^ ^]^ The second peak in the DSC graphs of the dried SPs occurred at ≈200 °C, which corresponds to the degradation of the polysaccharides.^[^ ^8^, ^29^ ^]^ Wang et al.^[^ ^30^ ^]^ reported that the pyrolysis of polysaccharides initiates with the random cleavage of glycosidic bonds, followed by further decomposition resulting in the formation of acetic and butyric acids, as well as various lower fatty acids, with a predominance of C2, C3, and C6. The difference in the width of the DSC graph among the dried SPs may be attributed to variations in moisture content and the structure of the polysaccharides.^[^ ^31^ ^]^ Hu et al.^[^ ^14^ ^]^ observed that different drying methods led to changes in the DSC profiles of the samples, primarily due to their effects on the free water content within the polysaccharide structure. However, Chen et al.^[^ ^32^ ^]^ reported that four drying methods, namely hot air drying, vacuum drying, freeze drying, and spray drying, did not show significant differences on the thermal stabilities of the samples.

#### 2.3.3 X‐Ray Diffraction (XRD)

**Figure 3:**
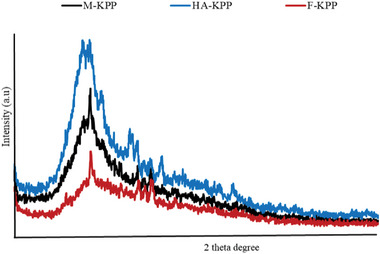
XRD graph of dried sulfated polysaccharides from Skipjack tuna skin using different drying methods.

Due to the direct impact of the crystalline or noncrystalline characteristics of samples on the physical properties of polysaccharides such as tensile strength, flexibility, solubility, and swelling, it is essential to evaluate the structural properties of polysaccharides.^[^ ^33^, ^34^ ^]^ Therefore, XRD analysis was employed to examine the structures of the dried SPs and confirm their crystalline nature. The XRD patterns of the obtained polysaccharides are presented in **Figure** **3**. As observed in the figure, all dried SPs exhibited only an amorphous peak at 20°, indicating that they were either amorphous polymers or semi‐crystalline materials. These findings may be attributed to the influence of the drying method on the complex composition and/or conformation of the molecules, which in turn affects the structure of the polysaccharides. Among the samples, the freeze‐dried SP exhibited the weakest intensity, suggesting a lower degree of crystallinity in F‐KPP.^[^ ^32^ ^]^ This could potentially be attributed to the higher temperature, which might have accelerated the aggregation process of the polysaccharides.^[^ ^32^ ^]^ This result aligns with the findings of Li et al.,^[^ ^35^ ^]^ who investigated the impact of the drying method on the physicochemical properties and antioxidant activities of *Hohenbuehelia serotina* polysaccharides.

### 2.4 Functional Properties

#### 2.4.1 Foam Properties

**Figure 4:**
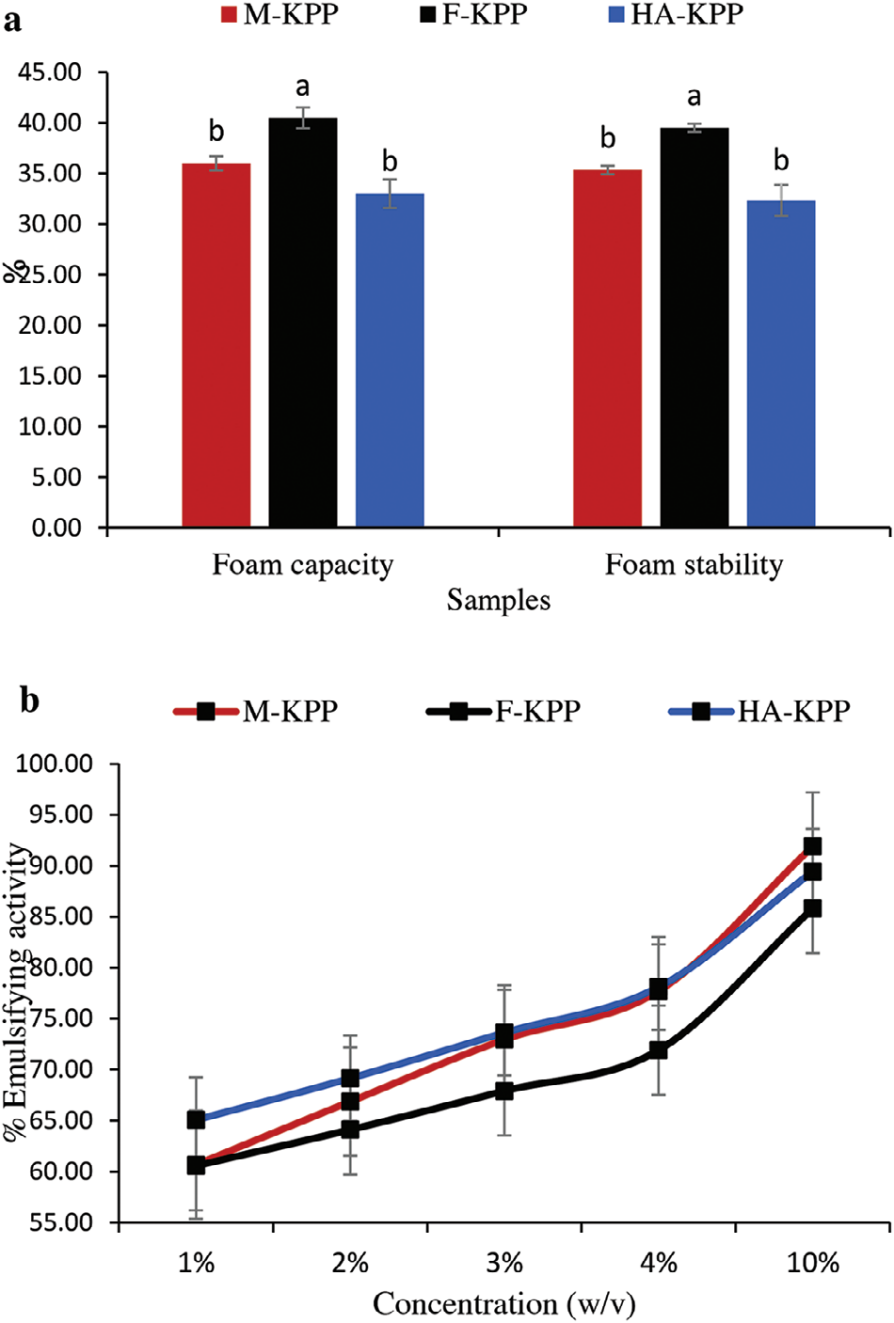
Foam (a), and Emulsifying Properties (b) of dried sulfated polysaccharides from Skipjack tuna skin using different drying methods.

Polysaccharides are widely used in food formulations and cosmetics as thickeners and stabilizers due to their hydrophilic characteristics.^[^ ^36^ ^]^ In food products like ice cream and milkshakes, a foaming agent is necessary to ensure proper gas distribution.^[^ ^36^ ^]^ **Figure** **4**a illustrates that different methods of processing dried sulfated polysaccharides yield varying foam capacities and stability. The results indicate that F‐KPP exhibited the highest foam capacity (40.50 ± 0.41%) and foam stability (39.50 ± 0.41%) (*P* < 0.05). Today, it is widely recognized that the foaming properties of polysaccharides are influenced by factors associated with the extraction process.^[^ ^3^, ^26^ ^]^ As the drying process is regarded as the concluding stage of extraction, the impact of this process is also well understood.^[^ ^36^, ^37^ ^]^ In the present study, as each of the drying methods has impacted the molecular weight of the dried samples, and considering that foaming properties are closely associated with the molecular weight and concentration of polysaccharides, it can be inferred that the polysaccharide with a lower molecular weight (F‐KPP in the present work) exhibits superior foaming properties. Also, Wang et al.^[^ ^37^ ^]^ conducted a study to assess the impact of various drying methods on the functional properties of flaxseed gum powders. They found that the choice of drying procedure had a significant effect on the foaming properties of the isolated samples. Furthermore, similar findings reported by Qin et al.^[^ ^36^ ^]^

#### 2.4.2 Emulsifying Properties

An emulsion refers to a mixture of two or more liquids that are typically immiscible.^[^ ^3^ ^]^ Figure 4b presents a comparison of the emulsifying activity of dried‐SPs. It is observed that all dried‐SPs exhibited a concentration‐dependent behavior. Notably, the highest emulsifying properties were observed in M‐KPP at a concentration of 10% (P < 0.05). Recent studies have highlighted the favorable surface activity and excellent emulsifying properties of polysaccharides derived from plants and algae, which are attributed to their specific sources.^[^ ^3^, ^38^ ^]^ Furthermore, previous research has demonstrated that the potent emulsifying properties are primarily influenced by functional groups such as carboxyl, sulfate, and hydroxyl groups, which are attached to hydrophobic proteinous moieties.^[^ ^38^, ^39^ ^]^ In a previous study conducted by Wang et al.,^[^ ^45^ ^]^ it was found that freeze drying and oven drying did not significantly alter the emulsion activity index (EAI) compared to the untreated flaxseed gum powders. However, spray drying was the only drying method that resulted in a reduction in the EAI value. Also, Qin et al.^[^ ^43^ ^]^ conducted a study to examine the impact of different drying methods on the emulsifying properties of pectin. They found that the sample dried using subcritical DME dehydration exhibited higher emulsifying properties compared to the samples dried using sun drying and freeze‐drying. The researchers attributed these results to the elevated protein content present in the subcritical DME dehydrated sample

### 2.5 Antioxidant Activities

#### 2.5.1 DPPH Scavenging Activity

**Figure 5:**
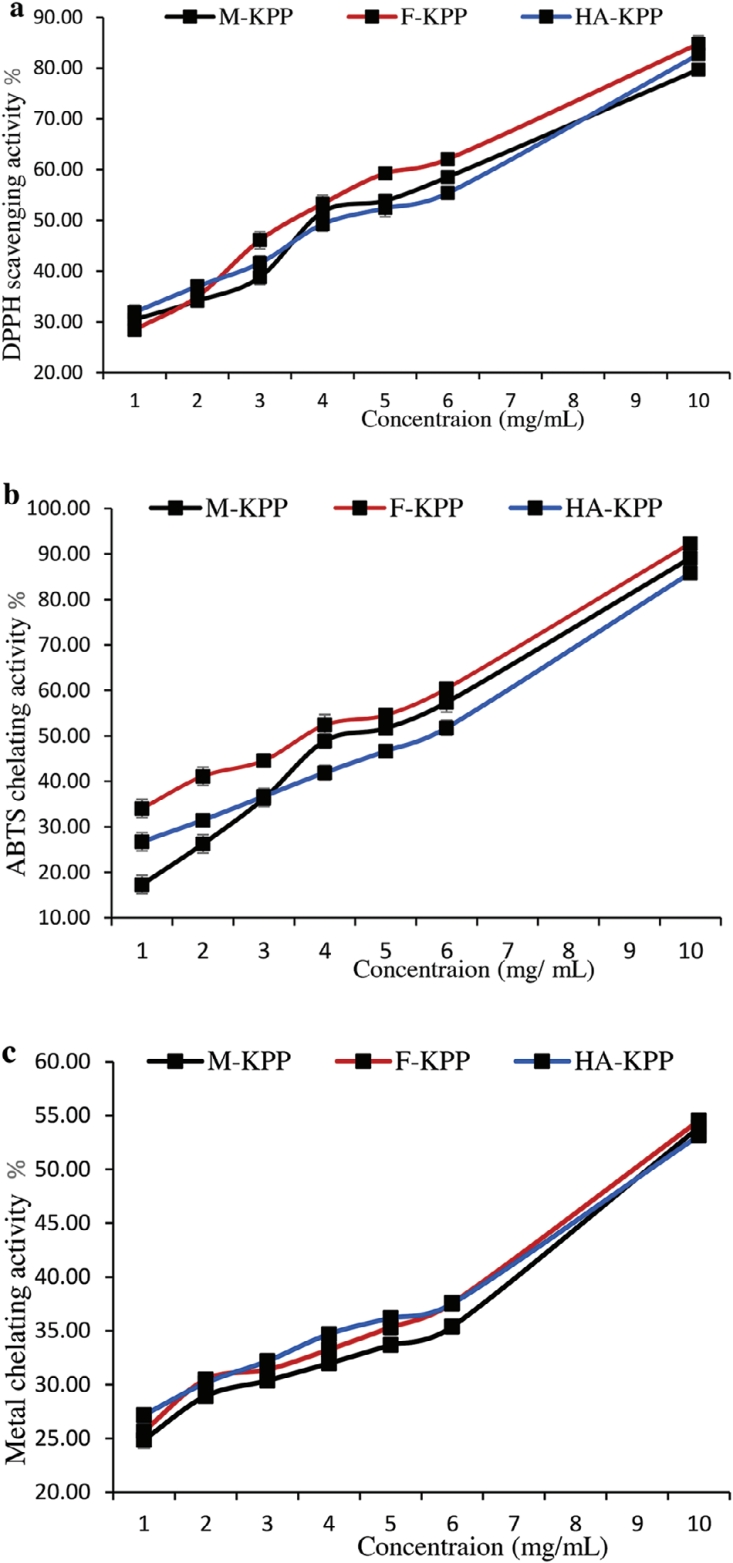
DPPH free radical‐scavenging (a), ABTS scavenging (b) and reducing power (c) activities of dried sulfated polysaccharides from Skipjack tuna skin using different drying methods.

The DPPH free radical is a stable radical widely used to assess the ability of samples to supply protons.^[^ ^13^ ^]^ **Figure** **5**a illustrates the scavenging activity of all dried SPs on DPPH radicals. At a concentration of 1 mg mL^−1^, M‐KPP, HA‐KPP, and F‐KPP displayed DPPH scavenging activities of 30.46%, 31.90%, and 28.45%, respectively. Furthermore, the DPPH scavenging activity of all samples increased with higher concentrations, with F‐KPP exhibiting the highest DPPH scavenging activity (84.77%) at a concentration of 10 mg mL^−1^. According to Zhao et al.,^[^ ^40^ ^]^ the antioxidant capacity of polysaccharides can be influenced by factors such as monosaccharide content, molecular weight, and conformation of the polysaccharides. Due to the highest content of sulfate, uronic acid, and protein in sample F‐KPP, it was expected that this sample would exhibit the highest antioxidant capacity in the DPPH test, and this result was also achieved at the high concentrations used in this test.^[^ ^32^ ^]^ Although at low concentrations, it showed a very slight difference compared to other samples, which may be due to the presence of varying amounts of active compounds in this test. However, considering the lower molecular weight of sample F‐KPP compared to other samples, the results obtained are also confirmed. Chen et al.,^[^ ^32^ ^]^ through their examination of the relationship between sulfate content, uronic acid, protein, and monosaccharides in *Chimonobambusa quadrangularis* polysaccharides dried using various methods, demonstrated a significant correlation between uronic acid and protein content, monosaccharide composition, and all evaluated antioxidant indices. They concluded that the antioxidant properties of the polysaccharide obtained through different drying processes were influenced by a combination of multiple factors rather than a single factor. Additionally, several studies have shown that the choice of drying method can impact the chemical composition and physicochemical structure of polysaccharides, thereby affecting their antioxidant properties.^[^ ^13^, ^41^ ^]^ For example, Wu et al.^[^ ^23^ ^]^ found that polysaccharides dried using the freeze‐drying method exhibited a higher DPPH scavenging rate compared to those dried using vacuum drying and air‐drying methods. They suggested that these results could be attributed to the impact of the drying process on the chemical changes in the polysaccharides. Similarly, Ma et al.^[^ ^26^ ^]^ reported similar findings, with freeze‐dried samples demonstrating the highest DPPH scavenging activity compared to samples dried using hot air drying and vacuum drying methods. Furthermore, Fan et al.^[^ ^41^ ^]^ obtained consistent results when using freeze‐drying, hot air drying, and vacuum drying for polysaccharides extracted from *Ganoderma lucidum*. According to Zhao et al.,^[^ ^40^ ^]^ the antioxidant capacity of polysaccharides may be linked to factors such as monosaccharide content, molecular weight, and conformation.

#### 2.5.2 ABTS Scavenging Activity

The results of the ABTS radical scavenging activity are presented in Figure 5b. All dried SPs showed significant scavenging of the ABTS radicals in a dose‐dependent manner. The highest ABTS radical scavenging activity was observed at a concentration of 10 mg mL^−1^, with the following order of activity from high to low: F‐KPP > M‐KPP > HA‐KPP. Various factors such as purity, molecular size, monosaccharide composition, structure, and conformation can influence the antioxidant activity of polysaccharides.^[^ ^34^, ^42^ ^]^ Therefore, the results obtained in this study can be explained by the lower molecular weight of the freeze‐dried SP compared to the others.^[^ ^34^ ^]^ Additionally, it has been reported that high uronic acid content in polysaccharides can alter their physiochemical properties and solubility, thereby affecting their antioxidant activities.^[^ ^8^, ^34^ ^]^ Wu et al.,^[^ ^23^ ^]^ evaluated the effects of drying methods on the antioxidant properties of *Agaricus blazei* polysaccharides and found that samples dried using the freeze‐drying method exhibited higher ABTS scavenging activity compared to samples dried using the hot air‐drying method. Also, Hu et al.,^[^ ^14^ ^]^ investigated the influence of drying methods on the antioxidant potential of polysaccharides from *Crassostrea gigas* and their results indicated that samples dried by the freeze‐drying method had lower scavenging activity compared to those dried by the spray‐drying and rotary evaporation‐drying methods.

#### 2.5.3 Reducing Power Activity

Figure 5c illustrates the reducing power activity of polysaccharides from Skipjack tuna skin dried using different methods. The reducing ability of all dried SPs increased as the concentration used increased. Among the dried polysaccharides tested at the highest concentration, those dried using a freeze dryer (F‐KPP) and hot air (HA‐KPP) exhibited the highest (54.53%) and lowest (53.15%) metal chelating activity, respectively, with no significant difference observed (Figure 4b). Fan et al.^[^ ^41^ ^]^ investigated the effects of various drying methods, including freeze drying, vacuum drying, and hot air drying, on the antioxidant activities of polysaccharides extracted from *Ganoderma lucidum*. Their results demonstrated that samples dried using the freeze‐drying method had significantly higher chelating activity compared to samples dried using vacuum drying or hot air drying, which aligns with our findings. In another study, Yuan et al.^[^ ^10^ ^]^ documented that polysaccharides from *Abelmoschus esculentus* dried using the freeze‐drying method exhibited higher antioxidant activities than those dried using hot air drying and vacuum drying methods. However, the results reported by Ma et al.^[^ ^26^ ^]^ reported contrasting results, stating that the reducing power activity of freeze‐dried polysaccharides obtained from the mushroom *Inonotus obliquus* was lower than those dried using hot air and vacuum drying methods.

### 2.6 Antibacterial Activity

Nowadays, with the increasing resistance of bacteria to commercial antibiotics, there is a growing need to search for alternative sources. Polysaccharides are among the natural compounds being considered.^[^ ^43^ ^]^ The Agar diffusion technique was used to evaluate the bacterial activity of dried SPs at concentrations of 10 and 20 mg mL^−1^. The results, shown in **Table** **3**, revealed that the antibacterial activity of dried‐SPs was higher at the concentration of 20 mg mL^−1^ compared to 10 mg mL^−1^. Additionally, there was a significant difference in the antibacterial activities of the samples at the same concentration. At a concentration of 10 mg mL^−1^, F‐KPP exhibited the highest inhibition zone against *L. monocytogenes*, *E. coli*, and *S. enterica*, while M‐KPP showed the highest inhibition activity against *S. aureus*. Among the tested bacteria, *S. enterica* was the most resistant with an inhibition zone of 6.6 mm, while *S. aureus* was the least resistant with a non‐growth zone of 26 mm. Importantly, at a concentration of 20 mg mL^−1^, M‐KPP demonstrated the highest antibacterial properties against all the investigated bacteria. Previous research conducted by Abdelhedi et al.^[^ ^33^ ^]^ showed that their polysaccharide extracted from common smooth hound had a greater inhibitory effect on Gram‐negative bacteria compared to Gram‐positive bacteria. Studies have generally suggested that the antibacterial activity of polysaccharides could be attributed to the disruption of bacterial cell walls and cytoplasmic membranes.^[^ ^24^, ^43^ ^]^ Furthermore, Hajji et al.^[^ ^43^ ^]^ proposed that polysaccharides could act as barriers, inhibiting bacterial growth by preventing the import of nutrients.

**Table 3:** Antibacterial activities of dried sulfated polysaccharides from Skipjack tuna skin using different drying methods.

Bacteria strains	M‐KPP		F‐KPP		HA‐KPP	
	Concentration [mg mL^−1^]		Concentration [mg mL^−1^]		Concentration [mg mL^−1^]	
	10	20	10	20	10	20
*L. monocytogenes*	10.0 ± 1.63^a^	22.3 ± 1.24^A^	11.3 ± 1.70^a^	18.3 ± 0.47^B^	7.6 ± 0.81^b^	15.0 ± 0.81^C^
*S. aureus*	15.0 ± 0.81^a^	24.6 ± 1.70^A^	8.3 ± 0.47^b^	14.3 ± 0.94^B^	7.6 ± 0.47^b^	13.6 ± 0.47^B^
*E. coli*	12.6 ± 0.94^a^	26.0 ± 0.81^A^	13.3 ± 1.24^a^	17.0 ± 0.81^B^	9.6 ± 0.94^b^	14.0 ± 0.81^C^
*S. enterica*	6.6 ± 1.24^b^	19.3 ± 1.24^A^	12.3 ± 0.47^a^	19.2 ± 0.11^A^	8.6 ± 0.47^b^	13.6 ± 0.47^B^

Different lowercase letters indicate significant differences in 10 mg mL^−1^ concentration between different SPs.

Different capital letters indicate significant differences in 20 mg mL^−1^ concentration between different SPs.

* Inhibition zones expressed based on mm. The values illustrate the means of three replicates ± standard deviations.

## 3 Conclusion

In this study, different drying technologies were used to systematically evaluate Skipjack tuna (*Katsuwonus pelamis*) skin. The study found that the drying techniques employed had an impact on the physicochemical characteristics and biological activities of the isolated sulfated polysaccharides. HA‐KPP, M‐KPP, and F‐KPP samples showed low molecular weight distributions, with F‐KPP having the highest sulfate content, followed by HA‐KPP and M‐KPP. Moreover, all sulfated polysaccharides (SPs) exhibited significant antioxidant capacities and antibacterial activities in vitro. Particularly, F‐KPP demonstrated notably higher antioxidant and foaming activities compared to the other samples. These findings provide valuable insights for selecting suitable drying methods when processing isolated sulfated polysaccharides obtained from fish.

## 4 Experimental Section

### Fish By‐Product Collection

The skins of *K. pelamis* were obtained from a nearby company in Babolsar, Mazandaran, Iran. To ensure their freshness, the skins were covered with ice in a ratio of 1:3 and transported to the Seafood Processing Laboratory at Tarbiat Modares University. Once at the laboratory, the skin samples were carefully cleaned and then stored at −20 °C until used for extracting the sulfated polysaccharides (SPs).^[^ ^44^ ^]^

### Bacterial Strains

The antibacterial activity of SPs was examined against two Gram‐positive (*Staphylococcus aureus* and *Listeria monocytogenes*) and two Gram‐negative (*Escherichia coli* and *Salmonella enterica*). These microorganisms used for the assay were received from the Pasteur Institute of Iran.

### Extraction of Polysaccharides

The polysaccharides were extracted from the *K. pelamis* skins using an ethanol precipitation procedure as described by Naghdi et al.^[^ ^3^ ^]^ Initially, 100 g of chopped skins were combined with 100 mL of distilled water. The mixture was then heated at 95 °C for 15 min to deactivate the endogenous enzymes. After cooling, Alcalase (pH 7.5 at 50 °C) was added to the mixture at a concentration of 500 U g^−1^ of samples for enzymatic proteolysis. The mixture was stirred and left for 12 h at 50 °C. Following the extraction, the Alcalase was inactivated by heating the mixture at 95 °C for 15 min and then placing it in an ice bath. The supernatant was obtained by centrifuging the mixture at 2800 ×g for 30 min at 4 °C. The sulfated polysaccharides (SPs) present in the collected supernatant were precipitated using absolute ethanol (v/2v) at 4 °C for 12 h. The precipitated SPs were then collected by centrifugation at 2800 ×g for 30 min at 4 °C. Finally, the obtained crude polysaccharides were dried using different drying protocols.

### Drying Experiments of *K. pelamis* Skin Polysaccharides

After extracting the sulfated polysaccharides from the skins of *K. pelamis*, the resulting sample underwent drying using three different techniques: microwave drying, freeze drying, and hot air drying. In the microwave drying method, the sample was placed in a petri dish and transferred to a Microwave oven (LG MG‐2313, South Korea) set at a microwave power of 600 W for 10 min.^[^ ^10^, ^19^ ^]^ For the freeze‐drying technique, the samples were dried at −40 °C for 48 h using a freeze dryer (FD‐5003‐BT, Mall Kala, Iran). The hot air‐drying method involved the use of an Oven (Memmert UNB 100, Germany), set at a temperature of 45 °C for 72 h. Throughout this process, the samples were thoroughly mixed every 12 h to ensure uniform drying. Finally, the dried polysaccharides obtained from the microwave, freeze, and hot air‐drying techniques were respectively labeled as M‐KPP, F‐KPP, and HA‐KPP, and were utilized for further analysis.

### Chemical Composition of SPs

The phenol–sulfuric acid method at 490 nm with D‐ fucose as the standard used to determine the total carbohydrate content.^[^ ^45^ ^]^ To measure the content of protein in different extracts, Lowry method with bovine serum albumin as the standard was used.^[^ ^46^ ^]^ The BaCl_2_ gelatine method at 360 nm was used to determine the content of total sulfate.^[^ ^47^ ^]^ The m‐hydroxybiphenyl method at 525 nm with D‐glucuronic acid as the standard was applied to measure uronic acid content.^[^ ^48^ ^]^

### Monosaccharide Compositions of SPs

The monosaccharide composition of sulfated polysaccharides (SPs) was determined using the method outlined by Naghdi et al.^[^ ^49^ ^]^ In summary, 5 mg of each SP was subjected to hydrolysis with 2 m trifluoroacetic acid at a temperature of 121 °C for a duration of 2 h. The resulting hydrolysates were then analyzed using GC‐MAS (gas chromatography mass spectrometry). The monosaccharide standards employed in this analysis included galacturonic acid (Gal A), glucuronic acid (Glc A), mannose, rhamnose, and xylose. The findings were reported in terms of the relative area of the peaks.

### Molecular Weight

The molecular weight of the extracted sulfated polysaccharides (SPs) was determined using an HPSEC–UV–MALLS–RI system, which consists of a high‐performance size exclusion chromatography column coupled with UV, multi‐angle laser light scattering, and refractive index detection. The sample preparation and calculation of the average molecular weight (Mw) of the SPs followed the methodology previously described by Alboofetileh et al.^[^ ^6^ ^]^

### Structural Characterization of SPs

*Fourier Transform Infrared (FTIR) Spectroscopy Analysis of SPs*: Fourier‐transform infrared spectroscopy (FTIR) pattern of samples was determined using a Horiba FT‐730 spectrometer (Bruker Instruments, Billerica, USA). In summary, dried samples (2 mg) were combined with KBr powder (100–200 mg) and packed into light discs. The spectra (4000–400 cm^−1^) were recorded with a resolution of 4 cm^−1^ and 64 scans.^[^ ^4^ ^]^

*Differential Scanning Calorimetry (DSC)*: Differential scanning calorimetry (DSC) supplied with a mechanical cooling system was employed to evaluate the thermal behavior of the crude polysaccharide. The experiments were completed over a temperature range of −50–430 °C at a rate of 30 °C min^−1^ in a nitrogen flow rate of 20 mL min^−1^.^[^ ^31^ ^]^

*X‐Ray Diffraction of SPs*: The crystallinity of the samples was determined by employing an X‐ray powder diffractometer (Brüker, Germany) at a scattering angle range of 2°–80° and with a step size of 0.02° and a counting time of 5 s step^−1^.^[^ ^50^ ^]^

### Functional Properties

Foam Properties: SPs were evaluated for foam capacity (FC) and foam stability (FS) according to a protocol described by Naghdi et al.^[^ ^49^ ^]^ In this method, 5 mL of 1% w/v SP solutions were homogenized at room temperature for 3 min at 2000 rpm. After whipping the solution, the foam stability was determined by allowing it to stand undisturbed at room temperature for 30 min.

FC and FS were determined as follows:

(1) $$\mathrm{FC}\left(\%\right)=\left(VT-V0\;/\;V0\right)\times 100$$

(2) $$\mathrm{FS}\left(\%\right)=\left(Vt-V0\;/\;V0\right)\times 100$$

After whipping, VT equals the volume after whipping, V0 equals the volume before whipping, and Vt equals the volume after 30 min at room temperature.

*Emulsifier Properties*: To evaluate the emulsifying properties of the SPs, sunflower oil was used. Solutions of SPs at concentrations of 1%, 2%, 3%, 4%, and 10% (w/v) were dissolved in the sunflower oil and vigorously vortexed for 2 min.^[^ ^3^ ^]^ The mixture was left for 24 h, then calculated the emulsification index (E24) using the following equation:

(3) $$E24=\left(He/Ht\right)\times 100$$

Ht and He are the emulsion layer heights and the mixture total heights, respectively.

### Determination of Antioxidant Activities of SPs

*DPPH Scavenging Activity*: The DPPH radical scavenging activity of the dried sulfated polysaccharides (SPs) was assessed using a method previously described by Naghdi et al.^[^ ^3^ ^]^ In this method, a 2 mL solution of 0.1 mM DPPH radical was combined with a 2 mL water solution containing different concentrations of polysaccharides (1, 2, 3, 4, 5, 6, and 10 mg). The mixture was vigorously shaken and kept in the dark for 30 min. Afterward, the absorbance was measured at 517 nm. The DPPH radical scavenging activity of the SPs was determined using the following equation:

(4) $$\mathrm{DPPH}\mathrm{scavenging}\mathrm{activity}\%=\left[\mathrm{Ac}-\mathrm{As}/\mathrm{Ac}\right]\times 100$$

where Ac is the absorbance of the control (100 µL of ethanol with 100 µL of the DPPH solution) and As the absorbance of sample solutions.

*ABTS Scavenging Activity*: The ability of dried sulfated polysaccharides (SPs) to scavenge ABTS radicals was evaluated using a modified version of the procedure reported by Wu et al.^[^ ^17^ ^]^ with some modifications. To generate the ABTS radical cation, 5 mL of ABTS solution (7 mm) was mixed with 1 mL of potassium persulfate (15 mm) and incubated in the dark at room temperature for 24 h. After the incubation period, the ABTS solution was diluted with deionized water until the absorbance reached 0.70 (±0.02) at 734 nm. Then, 0.05 mL of different concentrations of polysaccharides were added to 0.2 mL of the diluted ABTS solution and incubated for 15 min at 20 °C. Finally, the absorbance of the mixture was measured at 734 nm using a Multimode Reader spectrophotometer from Thermo, USA. The ABTS radical scavenging activity was calculated using the following equation:

(5) $$\mathrm{ABTS}\;\mathrm{scavenging}\;\mathrm{activity}\;\left(\%\right)=\left(\mathrm{Ac}-\mathrm{As}\right)/\mathrm{Ac}\times 10$$

in which here, Ac was the absorbance of control, As was the absorbance of polysaccharide sample solution.

*Reducing Power*: The test was conducted according to the method described by Jridi et al.^[^ ^22^ ^]^ The SP solutions prepared in different concentrations of 0.5, 1, 1.5, and 2 mg mL^−1^ were added to 100 to 50 µL of 2 mm FeCl_2_ and 450 µL of distilled water, respectively. The mixtures were left at room temperature for 5 min, and then 200 µL of 5 mm ferrozine solution was added to initiate the reaction. The mixture was also kept at room temperature for 10 min after shaking. To calculate the chelating activity (%) of different solutions, the absorbance at 562 nm was measured for each solution as follows:

The percent of ferric reducing power of samples was calculated according to the following equation:

(6) $$\mathrm{Metal}\;\mathrm{chelating}\;\mathrm{activity}\;\left(\%\right)=\left[\left(\mathrm{ODC}+\mathrm{ODB}-\mathrm{ODS}\right)/\mathrm{ODC}\right]\times 100$$

where ODC, ODB and ODS indicate the absorbance of the control, the blank and the sample reaction tubes, respectively. The experiments were done in triplicate.

### Antibacterial Activity

*Agar Diffusion Method*: The agar diffusion method, described by Naghdi et al.,^[^ ^3^ ^]^ was used in this study. In summary, culture suspensions of each bacteria (200 µL), with an absorbance of 0.08 at 600 nm, were evenly spread on Trypticase soy agar using a sterile swab. Then, different solutions of sulfated polysaccharides (SPs) (25, 50, and 100 mg mL^−1^ in distilled water) were added to cleaned wells (6 mm in diameter) created in the agar. The Petri dishes were incubated at 37 °C for 24 h. The antimicrobial activity was evaluated by measuring the diameter of the inhibition zone around the wells in millimeters. This process was repeated three times for each trial to ensure accuracy.

### Statistical Analysis

Statistical analyses were performed using SPSS ver. 22.0. One‐way analysis of variance (ANOVA) and Duncan's multiple range test were utilized to determine significant differences between the variables. Differences were considered significant at a p‐value of less than 0.05. The results were expressed as a mean value of three replicates ± SD (n = 3).

## Conflict of Interest

The authors declare no conflict of interest.

## Data Availability

Research data are not shared.

## References

[1] S. Pezeshk , M. Rezaei , H. Hosseini , M. Abdollahi , Food Hydrocoll. 2021, 118, 106768.

[2] A. O. of the U. N. F. Department, The state of world fisheries and aquaculture, Food and Agriculture Organization of the United Nations, 202AD.

[3] S. Naghdi , M. Rezaei , M. Tabarsa , M. Abdollahi , Food Bioprocess Technol 2023, 16, 1258.

[4] M. Jridi , M. Mezhoudi , O. Abdelhedi , S. Boughriba , W. Elfalleh , N. Souissi , R. Nasri , M. Nasri , Carbohydr. Polym. 2018, 194, 319. 29801845 10.1016/j.carbpol.2018.04.038

[5] T. Yang , M. Jia , S. Zhou , F. Pan , Q. Mei , Int. J. Biol. Macromol. 2012, 50, 768. 22155400 10.1016/j.ijbiomac.2011.11.027

[6] M. Alboofetileh , M. Rezaei , M. Tabarsa , S. G. You , J. Food Process Eng. 2018, 42, e12979.

[7] F. A. Figueroa , R. T. Abdala‐Díaz , C. Pérez , V. Casas‐Arrojo , A. Nesic , C. Tapia , C. Durán , O. Valdes , C. Parra , G. Bravo‐Arrepol , Mar. Drugs. 2022, 20, 458. 35877751 10.3390/md20070458 PMC9317217

[8] S. Hu , G. Zhao , Y. Zheng , M. Qu , Q. Jin , C. Tong , W. Li , PLoS One 2017, 12, 1. 10.1371/journal.pone.0188536 PMC5703540 29176846

[9] W. Liu , F. Li , P. Wang , X. Liu , J. He , M. Xian , L. Zhao , W. Qin , R. Gan , D. Wu , Int. J. Biol. Macromol. 2020, 148, 1211. 31758998 10.1016/j.ijbiomac.2019.10.211

[10] Q. Yuan , Y. He , P. Y. Xiang , Y. J. Huang , Z. W. Cao , S. W. Shen , L. Zhao , Q. Zhang , W. Qin , D. T. Wu , Int. J. Biol. Macromol. 2020, 147, 1053. 31756490 10.1016/j.ijbiomac.2019.10.073

[11] H. M. Shang , H. Z. Zhou , R. Li , M. Y. Duan , H. X. Wu , Y. J. Lou , PLoS One 2017, 12, 1. 10.1371/journal.pone.0183001 PMC5570291 28837625

[12] H. Shang , H. Zhou , M. Duan , R. Li , H. Wu , Y. Lou , Int. J. Biol. Macromol. 2018, 112, 889. 29428386 10.1016/j.ijbiomac.2018.01.198

[13] L. Ma , H. Chen , W. Zhu , Z. Wang , Food Res. Int. 2013, 50, 633.

[14] G. Chen , Q. Hong , N. Ji , W. Wu , L. Ma , Int. J. Biol. Macromol. 2020, 155, 674. 32234437 10.1016/j.ijbiomac.2020.03.223

[15] W. Li , D.‐T. Wu , F. Li , R.‐Y. Gan , Y.‐C. Hu , L. Zou , Molecules 2021, 26, 4395. 34361549 10.3390/molecules26154395 PMC8347772

[16] H. Shang , H. Zhou , M. Duan , R. Li , H. Wu , Y. Lou , Int. J. Biol. Macromol. 2018, 112, 889. 29428386 10.1016/j.ijbiomac.2018.01.198

[17] S. Wu , F. Li , S. Jia , H. Ren , G. Gong , Y. Wang , Z. Lv , Carbohydr. Polym. 2014, 103, 414. 24528748 10.1016/j.carbpol.2013.11.075

[18] Y. Fu , K. Feng , S. Wei , X. Xiang , Y. Ding , H. Li , L. Zhao , W. Qin , R. Gan , D. Wu , Int. J. Biol. Macromol. 2020, 145, 611. 31887373 10.1016/j.ijbiomac.2019.12.226

[19] W. Liu , F. Li , P. Wang , X. Liu , J. J. He , M. L. Xian , L. Zhao , W. Qin , R. Y. Gan , D. T. Wu , Int. J. Biol. Macromol. 2020, 148, 1211. 31758998 10.1016/j.ijbiomac.2019.10.211

[20] S. Zuo , H. Ge , Z. Li , S. Wang , K. Yang , J. Yuan , Y. Yang , W. Jiang , Y. Zhang , ACS Food Sci. Technol. 2024, 4, 404.

[21] M. Alboofetileh , M. Rezaei , M. Tabarsa , S. G. You , F. Mariatti , G. Cravotto , Int. J. Biol. Macromol. 2019, 128, 244. 30684576 10.1016/j.ijbiomac.2019.01.119

[22] N. Souissi , S. Boughriba , O. Abdelhedi , M. Hamdi , M. Jridi , S. Li , M. Nasri , RSC Adv. 2019, 9, 11538. 35520239 10.1039/c9ra00959k PMC9063433

[23] R. A. Cave , S. A. Seabrook , M. J. Gidley , R. G. Gilbert , Biomacromolecules 2009, 10, 2245. 19627139 10.1021/bm900426n

[24] O. Abdelhedi , R. Nasri , N. Souissi , M. Nasri , M. Jridi , Carbohydr. Polym. 2016, 152, 605. 27516310 10.1016/j.carbpol.2016.07.048

[25] D. Yang , F. Lin , Y. Huang , J. Ye , M. Xiao , Int. J. Biol. Macromol. 2019, 155, 1003. 31712137 10.1016/j.ijbiomac.2019.11.064

[26] S. Ahmadi , M. Sheikh‐Zeinoddin , S. Soleimanian‐Zad , F. Alihosseini , H. Yadav , LWT 2019, 100, 1. 10.1016/j.lwt.2018.10.027 PMC6309185 35238861

[27] A. Ginzberg , E. Korin , S. Arad , Biotechnol. Bioeng. 2008, 99, 411. 17625787 10.1002/bit.21573

[28] L. Kong , L. Yu , T. Feng , X. Yin , T. Liu , L. Dong , Carbohydr. Polym. 2015, 125, 1. 25857953 10.1016/j.carbpol.2015.02.042

[29] D. Oliveira , A. Luis , L. De Araújo , P. Vieira , F. D. A. Rocha , Procedia Eng. 2017, 200, 193.

[30] L. C. Wang , L. Q. Di , R. Liu , H. Wu , Carbohydr. Polym. 2013, 92, 106. 23218272 10.1016/j.carbpol.2012.08.084

[31] F. Krichen , H. Bougatef , N. Sayari , F. Capitani , I. Ben Amor , I. Koubaa , F. Maccari , V. Mantovani , F. Galeotti , N. Volpi , A. Bougatef , Carbohydr. Polym. 2018, 197, 451. 30007634 10.1016/j.carbpol.2018.06.040

[32] G. Chen , C. Li , S. Wang , X. Mei , H. Zhang , J. Kan , Food Chem. 2019, 292, 281. 31054677 10.1016/j.foodchem.2019.04.060

[33] P. S. Saravana , Y. J. Cho , Y. B. Park , H. C. Woo , B. S. Chun , Carbohydr. Polym. 2016, 153, 518. 27561524 10.1016/j.carbpol.2016.08.014

[34] L. Jiang , W. Wang , P. Wen , M. Shen , H. Li , Y. Ren , Y. Xiao , Q. Song , Y. Chen , Q. Yu , J. Xie , Food Hydrocoll. 2020, 100, 105412.

[35] X. Li , L. Wang , Y. Wang , Z. Xiong , Process Biochem. 2016, 51, 1100.

[36] Z. Qin , H. M. Liu , X. C. Cheng , X. De Wang , Int. J. Biol. Macromol. 2019, 137, 801. 31255624 10.1016/j.ijbiomac.2019.06.209

[37] Y. Wang , D. Li , L. J. Wang , S. J. Li , B. Adhikari , Carbohydr. Polym. 2010, 81, 128.

[38] I. Trigui , H. Yaich , A. Sila , S. Cheikh‐Rouhou , A. Bougatef , C. Blecker , H. Attia , M. A. Ayadi , Int. J. Biol. Macromol. 2018, 117, 937. 29864536 10.1016/j.ijbiomac.2018.05.202

[39] A. Hamzaoui , M. Ghariani , I. Sellem , M. Hamdi , A. Feki , I. Jaballi , M. Nasri , I. Ben Amara , Int. J. Biol. Macromol. 2020, 148, 1156. 31917214 10.1016/j.ijbiomac.2020.01.009

[40] Q. Zhao , B. Dong , J. Chen , B. Zhao , X. Wang , L. Wang , S. Zha , Y. Wang , J. Zhang , Y. Wang , Carbohydr. Polym. 2015, 127, 176. 25965471 10.1016/j.carbpol.2015.03.041

[41] L. Fan , J. Li , K. Deng , L. Ai , Carbohydr. Polym. 2012, 87, 1849. 10.1016/j.carbpol.2012.06.013 22840014

[42] Q. Wang , Y. Zhao , X. Feng , S. A. Ibrahim , W. Huang , Y. Liu , J. Food Sci. Technol. 2021, 58, 3622. 34366479 10.1007/s13197-021-05120-6 PMC8292488

[43] M. Hajji , M. Hamdi , S. Sellimi , G. Ksouda , H. Laouer , S. Li , Carbohydr. Polym. 2019, 206, 380. 30553336 10.1016/j.carbpol.2018.11.020

[44] M. Jridi , R. Nasri , Z. Marzougui , O. Abdelhedi , M. Hamdi , M. Nasri , Int. J. Biol. Macromol. 2019, 123, 1221. 30465838 10.1016/j.ijbiomac.2018.11.170

[45] K. Dubois , K. Gilles , P. Hamilton , A. Rebers , F. Smith , Anal. Chem. 1956, 28, 350.

[46] O. H. Lowry , N. J. Rosebrough , A. L. Farr , R. J. Randall , J. Biol. Chem. 1951, 193, 265. 14907713

[47] F. A. Loyd , A. G. Dogson , K. S. Price , R. G. Rose , Biochem. Biophys. Acta 1960, 46, 108.

[48] T. Bitter , H. M. Muir , Anal. Biochem. 1962, 4, 330. 13971270 10.1016/0003-2697(62)90095-7

[49] S. Naghdi , M. Rezaei , M. Tabarsa , M. Abdollahi , Sustain. Chem. Pharm. 2023, 32, 101033.

[50] F. Krichen , W. Karoud , A. Sila , B. E. Abdelmalek , R. Ghorbel , S. Ellouz‐Chaabouni , A. Bougatef , Int. J. Biol. Macromol. 2015, 75, 283. 25647621 10.1016/j.ijbiomac.2015.01.044

